# Global metabolic profile and multiple phytometabolites in the different varieties of *Gastrodia elata* Blume

**DOI:** 10.3389/fpls.2023.1249456

**Published:** 2023-10-17

**Authors:** Xu Zeng, Jiaxue Li, Tongyao Chen, Yuanyuan Li, Shunxing Guo

**Affiliations:** Institute of Medicinal Plant Development, Chinese Academy of Medical Sciences and Peking Union Medical College, Beijing, China

**Keywords:** *Gastrodia elata*, variety, phytometabolite, aromatic compounds, HPLC fingerprints

## Abstract

*Gastrodia elata* Blume (Tianma in Chinese), a myco-heterotrophic orchid, is widely distributed in China. Tubers derived from this orchid are traditionally used as both medicinal and edible materials. At present, five primary varieties of *G. elata* are recorded in the “Flora of China.” Among them, the three main varieties currently in artificial cultivation are *G. elata* f. *elata* (GR, red stem), *G. elata* f. *glauca* (GB, black stem), and *G. elata* f. *viridis* (GG, green stem). In our study, the metabolic profiles and chemical composition of these three varieties were determined via UPLC-MS/MS and HPLC-UV. In total, 11,132 metabolites were detected, from which multiple phytometabolites were identified as aromatic compounds, heteroatomic compounds, furans, carbohydrates, organic acids, and their derivatives. A number of differentially expressed metabolites (DEMs) were annotated as bioactive ingredients. Overall, parishins, vanilloloside, and gastrodin A/B in the GB group were markedly higher, whereas gastrodin, gastrol, and syringic acid were more enriched in the GG or GR groups. Moreover, HPLC fingerprint analysis also found six metabolites used as markers for the identification of Gastrodiae Rhizoma in the Chinese Pharmacopoeia, which were also typical DEMs in metabolomics. Of these, gastrodin, 4-hydroxybenzyl alcohol, citric acid, and adenosine were quantitatively detected, showing a similar result with the metabolomic data. In summary, our findings provide novel insights into the phytochemical ingredients of different *G. elata* varieties, highlighting diverse biological activities and healthcare value.

## 1 Introduction

*Gastrodia elata* Blume (Tianma in Chinese) is a species of myco-heterotrophic herb in Orchidaceae. This orchid is a valuable traditional Chinese medicine that has been recorded in the ancient Chinese books “Shen Nong’s Classic of Materia Medica”. In this decade, the fresh tuber of *G. elata* has been considered an important healthcare food. This medicinal orchid has various pharmacological activities such as sedation, hypnosis, intelligence enhancement, anti-epileptics, analgesics, neuroprotective drugs, anti-depressants, cardiovascular protection, and immune enhancement (Zhan et al., 2016; Liu et al., 2018). A number of studies have exhibited that it had a strong potential for combating Alzheimer’s and Parkinson’s disease (Jang et al., 2015; Heese, 2020). Accordingly, *G. elata* is believed to possess several of the active components.

Records from “Flora of China” indicate that there are primarily five varieties of *G. elata*, which are widely distributed in Wumeng, Qinling, and Dabie mountains (Wu et al., 2009). They are *G. elata* f. *elata* (red stem, GR), *G. elata* f. *glauca* (black stem, GB), *G. elata* f. *viridis* (green stem, GG), *G. elata* f. *flavid* (yellow stem, GY), and *G. elata* f. *alba* (yellow-white stem, GYW). The numbers of wild *G. elata* have declined dramatically in recent years. Unique biological property, the damage of ecological environment, and over-exploitation were the main cause of wild resource reduction. Fortunately, artificial cultivation of *G. elata* has been developing in China, Japan, South Korea, and India for several decades (Guo et al., 2016). At present, there are three main cultivars in artificial cultivation: red Tianma (GR), black Tianma (GB), and green Tianma (GG). In general, *G. elata* varieties are primarily divided according to the characteristics in the stem and flower color. Of these, GR has the highest annual production, and GB has the highest price on market. That is mainly because GB was not only good in quality but also sweet in taste (Yu E. et al., 2022).

Over the past decade, numerous studies have reported on the major pharmaceutical application of a single variety of *G. elata* (Wu et al., 2023). Changes in compositional content have been related to cultivation area, fungal partner, and medicinal material processing, such as gastrodin and 4-hydroxybenzyl alcohol. Differences in chemical properties and biological activities among different varieties have not been widely investigated. From a recent study, polysaccharide characteristics were investigated among four different varieties of *G. elata* (Ji et al., 2022). Unfortunately, the multiple metabolites present in different *G. elata* are currently not described. Metabolomics is a rapidly developing technology that has effectively promoted plant sciences and drugs discovery. In this study, the global metabolic profile of three major *G. elata* varieties are investigated by UPLC-MS/MS-based metabolomics. Analyses and reviews of phytometabolites and their pharmacological effects were performed. In addition, HPLC was used for chromatographic fingerprint analysis and component examination. Our study has served as a foundation for the further development and use of different *G. elata* varieties.

## 2 Materials and methods

### 2.1 Samples

**Figure 1:**
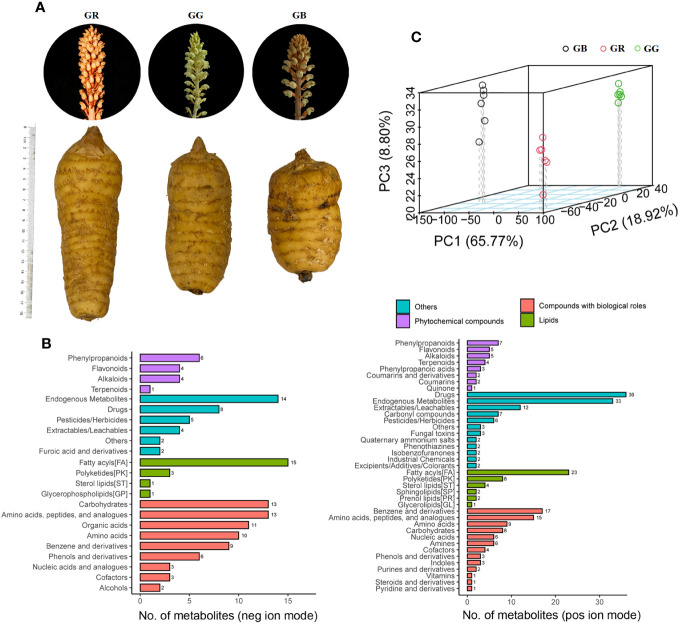
The tubers, stems, and flowers of three *G. elata* varieties **(A)**, classification of metabolites **(B)**, and PCA 3D score scatter plots **(C)**.

The samples of red Tianma (GR), black Tianma (GB), and green Tianma (GG) varieties (**Figure 1A**) were collected from the planting area (104.29492°E and 27.76122°N, and the altitude was 1,870 mm), Xiaocaoba town, Yiliang County, Yunnan Province, China. Subsequently, the fresh tubers were washed and cut into slices. After freeze drying, samples from each group were stored at −80°C.

### 2.2 Untargeted metabolomics

Sample treatment and metabolomics analyses were performed on six biological replicates from the GR, GB, and GG groups. BGI Genomics Co., Ltd. (Shenzhen, China) performed metabolite extraction and UPLC-MS/MS as described in the prior studies (Dunn et al., 2011; Di Guida et al., 2016). Briefly, 50 mg of freeze-dried powder was accurately weighed for extraction by ultrasonication using a precooling buffer for 30 min. Buffer formulation was as follows: 800 μL of 70% methanol and 20 μL of internal standard (0.3 mg/ml d3-Leucine, 13C9-Phenylalanine, d5-Tryptophan, and 13C3-Progesterone). Then, the mixture was maintained at −20°C for 60 min and centrifuged at 14,000 rpm for 15 min. Next, 600 μL of supernatant was collected for untargeted metabolomics. Moreover, quality control (QC) sample was composed of 20 μL of supernatant from each samples. During experimental process, the QC sample was included in the queue every six test samples to evaluate stability and repeatability.

The samples were separated on a UPLC system (Waters 2D, Milford, MA, USA) with hypersil GOLD aQ column (100 mm × 2.1 mm i.d., 1.9 μm; Thermo Fisher Scientific, USA). Mobile phase A and B were 0.1% aqueous formic acid and acetonitrile containing 0.1% formic acid, respectively. Chromatographic parameters were as follows: injection volume, 5.00 μL; column temperature, 40°C; flowrate, 0.35 mL/min; and elution gradient, 95% A (0 min–2 min), 95%–5% A (2 min–22 min), 5% A (22 min–27 min), and 95% A (27.1 min–30 min). The Thermo Fisher Scientific Q Exactive mass spectrometer was used for the identification of metabolites, which were ionized by electrospray ionization (ESI) in both positive and negative ion mode. Mass spectrometry parameters were as follows: stepped NCE, 20–40–60 eV; sheath gas flowrate, 40 arb; aux gas flowrate, 10 arb; spray voltage(|KV|) 3.80 (+) and 3.20 (−); capillary temperature, 320°C; aux gas heater temperature, 350°C; and scanning range, 100–1,500 m/z.

The raw data from UPLC-MS/MS were analyzed by Compound Discoverer 3.1 software (Thermo Fisher Scientific, USA). Subsequently, the metaX from BGI’s metabolomics software package was used for metabolite annotation, classification, and statistical analysis (Wen et al., 2017). In order to analyze the overall metabolic differences between groups, principal component analysis (PCA) was performed. Using VIP >1, p < 0.05 and fold change (FC) > 2 or < 0.5 value as threshold to identify differentially expressed metabolites (DEMs) based on the OPLS-DA and Student’s t-test. Metabolites were annotated by BGI High-Resolution Plant Metabolome Database, mzCloud database, Human Metabolome (http://www.hmdb.ca/), KEGG databases (using MetaboAnalyst, https://www.metaboanalyst.ca/), and 211 phytometabolites isolated from *G. elata* described in previous studies.

### 2.3 HPLC fingerprints analysis

The reference medicine materials of *G. elata* (purchased from National Institutes for Food and Drug Control) and freeze-dried samples were used for HPLC fingerprints analysis, as described in Chinese Pharmacopoeia 2020 edition and previous studies (Li et al., 2019; Commission, 2020). Briefly, 0.5 g of powder was ultrasonic extracted with 25 mL of 50% methanol for 30 min at 500 W and 40 kHz. After cooling, the filtrate was used for HPLC fingerprints analysis.

The HPLC system was composed of a 1260 HPLC system (Agilent Co., Milford, MA, United States) with Agela venusil ASB-C18 column (4.6 mm × 250 mm, 5 µm). Mobile phase A and B were acetonitrile and 0.1% phosphoric acid solution, respectively. Chromatographic parameters were as follows: injection volume, 3.00 μL; column temperature, 30°C; flowrate, 0.8 mL/min; detection wavelength, 220 nm; and elution gradient 3%–10% A (0 min–10 min), 10%–12% A (10 min–15 min), 12%–18% A (15 min–25 min), 18% A (25 min–40 min), and 18%–95% A (40 min–42 min). Chemstation software (Agilent) was used for data processing. Subsequently, Similarity Evaluation System for TCM chromatographic fingerprinting (2012 edition) were applied for evaluating the chromatographic profiles of the *G. elata* tuber.

### 2.4 Determination of gastrodin, 4-hydroxybenzyl alcohol, citric acid, and adenosine

Freeze-dried powder of test samples were used for the extraction of gastrodin and 4-hydroxybenzyl alcohol by the ultrasound-assisted method, as described in the Chinese Pharmacopoeia 2020 edition (Commission, 2020). In brief, 2 g of powder was weighed for extraction by ultrasonication using 50 mL ethyl alcohol for 30 min at 120 W and 40 kHz. After cooling, additional ethyl alcohol was added to compensate for the weight loss. Next, 10 mL of the supernatant fluid was concentrated, and ethanol was removed. The residue was further immersed with 25 mL of 3% acetonitrile. A 0.22-µm membrane was used to filter all the solution. Gastrodin and 4-hydroxybenzyl alcohol were determined by an external standard one-point method (50 and 25 μg/mL, respectively). The HPLC system consisted of a 1260 HPLC system (Agilent Co., Milford, MA, United States) with Agela venusil ASB-C18 column (4.6 mm × 250 mm, 5 µm). Mobile phase was acetonitrile–0.05% phosphoric acid solution (3:97, v:v). Chromatographic parameters were as follows: injection volume, 5.00 μL; column temperature, room temperature; flowrate, 0.8 mL/min; and detection wavelength, 220 nm.

Citric acid was extracted with by ultrasonics, as described in a previous study (Zhang et al., 2022). In brief, 1 g of freeze-dried powder of the test sample was extracted with 10 mL of 60% methanol for 30 min. After centrifugalizing, additional 60% methanol was added to compensate for the weight loss and filtered through a 0.22-µm membrane. The standard substance of citric acid was dissolved in 60% methanol to a concentration of 9 mg/mL. After diluting, the final concentrations of 0.1, 0.6, 0.9, 2, 3, 6, and 9 mg/mL were prepared (**Supplementary Figure S1**). The HPLC system consisted of a 1260 HPLC system (Agilent Co., Milford, MA, United States) with Akzo Nobel Kromasil 100-3.5-C18 column (4.6 mm × 150 mm, 3.5 µm). Mobile phase A and B were acetonitrile and 0.1% phosphoric acid solution, respectively. Chromatographic parameters were as follows: injection volume, 10.00 μL; column temperature, 30°C; flowrate, 1.0 mL/min; detection wavelength, 220 nm; and elution gradient, 15%–60% A (0 min–25 min).

The extract method of adenosine was similar with citric acid. The only difference between them was solvate. Adenosine was extracted using 10% methanol as solvate. The standard substance of adenosine was dissolved in water to a concentration of 500 μg/mL. After diluting, the final concentrations of 0.5, 1, 10, 25, 50, and 100 μg/mL were prepared (**Supplementary Figure S1**). The 1260 HPLC system (Agilent Co., Milford, MA, United States) with Akzo Nobel Kromasil 100-3.5-C18 column (4.6 mm × 150 mm, 3.5 µm) was used. Mobile phase A and B were acetonitrile and water, respectively. Chromatographic parameters were as follows: injection volume, 10.00 μL; column temperature, 30°C; flowrate, 1.0 mL/min; detection wavelength, 260 nm; and elution gradient, 10% A (0 min–5 min) and 90% A (5 min–10 min).

Chemstatio software (Agilent) was used for data processing. Student’s t-test for unpaired data by SPSS 19.0 was used for statistical analysis. A p-value < 0.05 was taken as significant.

## 3 Results and discussion

### 3.1 Metabolomics profiles

Plant secondary metabolites offer broad perspectives for application in the food and pharmaceutical industry. The metabolomics data set primarily consisted of secondary metabolites, which indicated differences in phytometabolites among the *G. elata* varieties. In the present study of fresh tubers from red Tianma (GR), black Tianma (GB), and green Tianma (GG), untargeted metabolomics was performed using UPLC-MS/MS approach. The MS1 intensity chromatograms of QC samples are presented in **Supplementary Figure S2**. After removing those that are redundant and noisy, a total of 11,132 spectral signals were detected by positive and negative ion modes (6,767 and 4,365, respectively, **Supplementary Table S1**). Metabolites were annotated by comparing MS1 molecular weight, MS2 fragment spectra, retention time, and whether reference standards existed. In total, there were 990 metabolites annotated in our metabolomics data from the *G. elata* varieties samples (**Supplementary Table S2**). Based on KEGG database, 173 and 110 metabolites were classified into 20 categories based on positive and negative ion modes, respectively (**Figure 1B**, **Supplementary Table S1**). Most phytometabolites were enriched in fatty phenylpropanoids, flavonoids, alkaloids, and terpenoids. The major lipid metabolites were grouped into fatty acyls, polyketides, and sterol lipids. Moreover, numerous metabolites were identified as carbohydrates, phenols, benzenes, amino acids, and peptides in the metabolomics data set.

**Figure 2:**
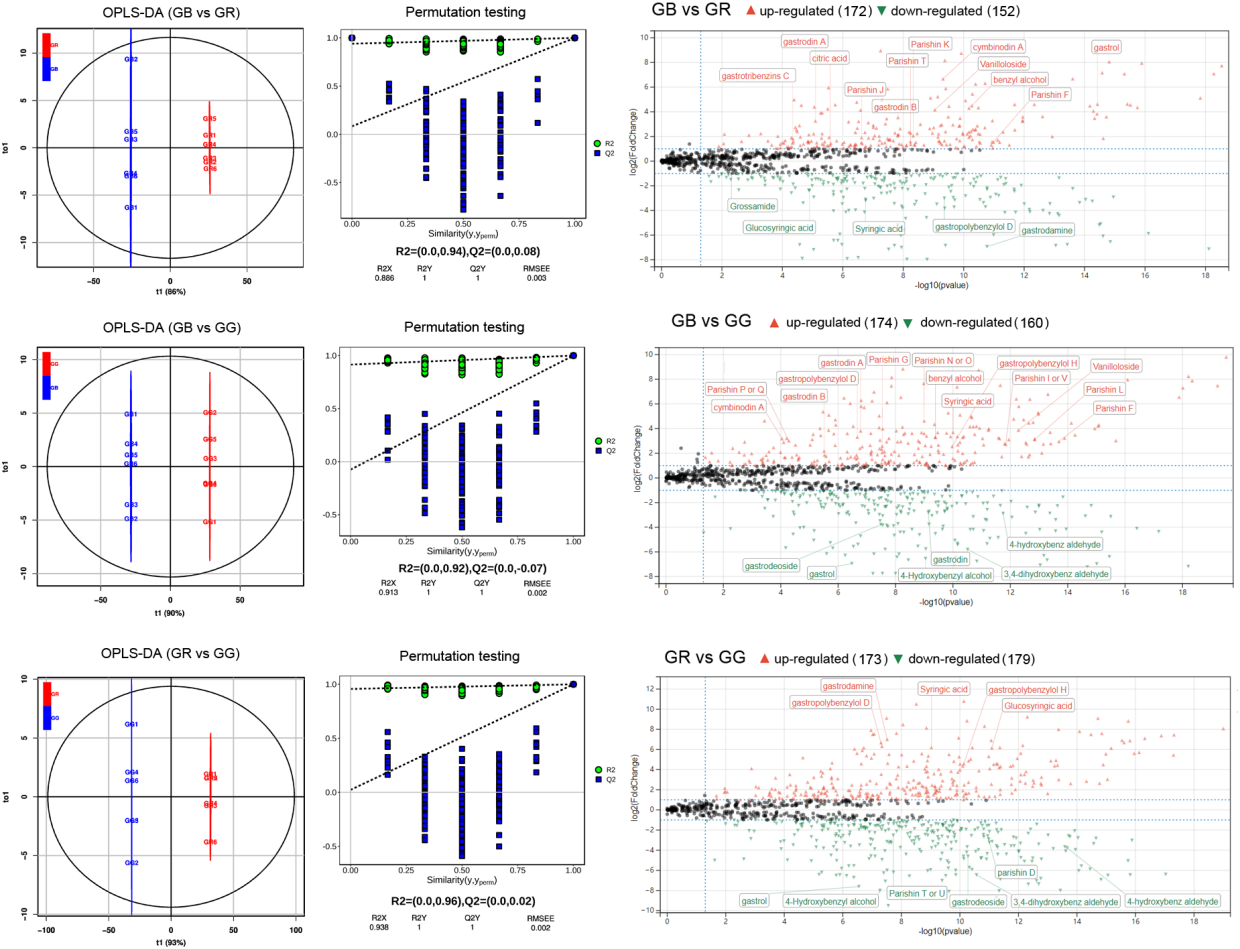
OPLS-DA score plots, permutation test, and volcano plot of differential metabolites between different *G. elata* varieties.

In addition to being a famous unsupervised pattern recognition method, PCA is also an important approach for reducing multi-dimensional data. **Figure 1C** shows the 3D score scatter plot of metabolomics profiles derived from *G. elata* varieties samples, with PC1 (x-axis), PC2 (y-axis), and PC3 (z-axis) accounting for 93.94% of the total variance. This result suggested that three *G. elata* varieties groups had large metabolic differences overall. In contrast, all of the GR (red dots), GB (black dots), and GG samples (green dots) were relatively concentrated, indicating small differences within the group. Meanwhile, OPLS-DA model, a supervised pattern recognition method, was used to further identify metabolite differences between groups. The score plot of three *G. elata* varieties is shown in **Figure 2**. The OPLS-DA score plot for each group showed strong clustering without overlap. The repeatability of OPLS-DA model was measured by permutation test (**Figure 2**). All of the goodness-of-fit parameter (R2) and the predictive ability parameter (Q2) were more than 0.9, indicating the repeatability of the model.

There was good separation of significantly differentially expressed metabolites (DEMs) between groups of *G. elata* varieties based on our criteria. When GB vs. GR, GB vs. GG, and GR vs. GG groups were compared, the 172, 174, and 173, and 152, 160, and 179 annotated metabolites (**Figure 2**) were identified as up- and down-accumulated metabolites, respectively. Taken together, numerous parishins (F, J, T, K, G, etc.), gastrodin A/B, and vanilloloside were up-accumulated metabolites in the GB group; gastropolybenzylol D, syringic acid, glucosyringic acid, and gastrodamine were more abundant in the GR group; and 4-hydroxybenzly alcohol, 3,4-dihydroxyben aldehyde, and gastrol were upregulated metabolites in the GG group.

### 3.2 Phytometabolites in *G. elata*

To date, there are more than 210 phytometabolites reported by the previous research on *G. elata* (Su et al., 2023), including aromatic compounds, furans, steroids, carbohydrates, organic acids and their esters, N-containing compounds, S-containing compounds, and their glycosides (Yu H. et al., 2022). Here, most of them were identified in the metabolomics data set from three *G. elata* varieties (**Supplementary Table S3**). Furthermore, aromatic compounds, the most abundant ingredients in *G. elata*, were composed of monobenzyl compounds, polyaromatic substituted glycosides, polybenzyl ethers, polybenzyl compounds, parishins, etc. As shown in **Supplementary Table S3**, 104 aromatic compounds, 2 furans, 4 carbohydrates, 11 organic acids and their esters, 11 N-containing compounds, 8 S-containing compounds, and 3 other metabolites were detected.

### 3.3 Aromatic compounds

**Figure 3:**
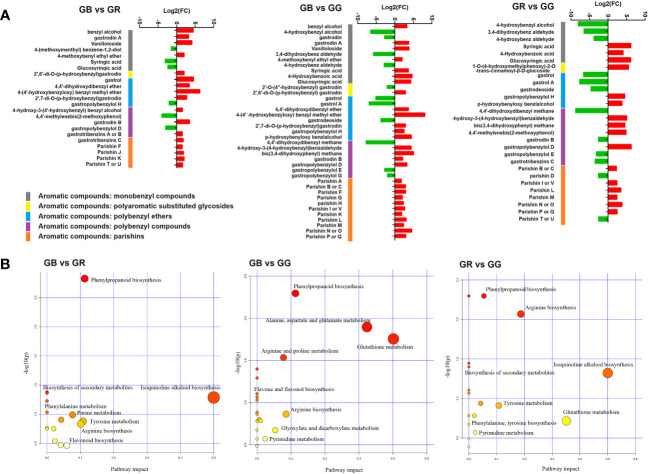
Fold change of differential metabolites annotated as aromatic compounds between different *G. elata* varieties **(A)** and KEGG pathway of DEMs between different *G. elata* varieties **(B)**.

Numerous reviews on *G. elata* have summarized the phytochemistry, compound structure, and quality control methods. Phenolics compounds and polysaccharides have been widely regarded as the typical and bio-active ingredients of *G. elata* (Shan et al., 2021). Among them, aromatic compounds are the main category. Recently, these aromatic compounds were further divided into eight subgroups on the basis of parent nucleus structure, connection modes, and subgroups function (Yu H. et al., 2022). DEMs annotated as aromatic compounds are shown in **Figure 3A**.

Monobenzyl compounds contain only one benzyl in the parent nucleus. It was widely reported that these constituents had neuroprotective effects, anti-inflammatory activity, anti-oxidant actions, and numerous other pharmaceutical functions. As shown in **Figure 3A** and **Supplementary Table S3**, there were 7, 11, and 6 monobenzyl compounds detected as DEMs between GB vs. GR, GB vs. GG, and GR vs. GG group, including gastrodin, 4-hydroxybenzyl alcohol, syringic acid, 4-hydroxybenz aldehyde, gastrodin A, and 6′-O-acetylgastrodin. In detail, gastrodin and 4-hydroxybenzyl alcohol, which are major bioactive constituents and two markers for the HPLC identification of Gastrodiae Rhizoma in the Chinese Pharmacopeia 2020 edition, were found to be up-accumulated in the GB group. The effects and mechanisms of gastrodin on the central nervous system disorders from preclinical models and the clinical evidence supporting pharmacological activities have been reviewed in a recent study (Liu et al., 2018). For example, an earlier study showed that gastrodin and 4-hydroxybenzyl alcohol exhibited potential anti-depressant effects in rats by downregulating monoamine metabolism and directly decreasing Slit1 expression (Chen et al., 2016). In addition, another study showed that 4-hydroxybenz aldehyde and vanilline (both more abundant in GG) derived from *G. elata* water extract reduced insulin resistance in diet-induced obese rats via inhibiting fat accumulation in adipocytes, promoting fat oxidation and increasing leptin signaling (Park et al., 2011). Moreover, benzyl alcohol, 4-methoxybenyl ethyl ether, and gastrodin A were more accumulated in GB. In summary, the results suggested that numerous monobenzyl compounds identified as DEMs were accumulated higher in the GG or GB groups.

Parishins are important ingredients in *G. elata*, which show several biological and pharmacologic effects. In the structure, the chemical bonds connecting the moieties of citric acid and gastrodin are ester linkages. The connections are easily broken by strong acidic or alkaline environment, enzymatic reaction, and continuous light (Tang et al., 2015). Our metabolomic analysis identified nearly all of the parishins recorded in the previous studies of *G. elata* (**Supplementary Table S3**). Among them, the relative contents of seven members, including parishin F, J, K, A, B, and H, was higher in the GB than in the GR or GG groups, whereas the relative content of parishin B/C, L, and M was lower in GG compared with GR group (**Figure 3A**). Parishins have been reported to possess unique bioactivity for the treatment of brain disorders such as anti-epileptic, anti-convulsive, sedative, and neuroprotective effects. A recent study reported that parishins exhibited the prevention of gut aging and the improvement of “leaky gut” in aged mice (Gong et al., 2023). Another study has shown that parishin J in *G. elata* could have protective effects on hypoxia/reoxygenation injury in H9c2 cardiomyocytes (Wang et al., 2020). Taken together, our results implied that various parishins were much more abundant in the GB than in the GR or GG groups.

Polyaromatic-substituted glycosides are novel compounds that have been detected in the past few years (Wang et al., 2019; Xu et al., 2019). In their structure, two or more hydroxy groups in a monosaccharide are substituted to aromatic groups. At present, only nine polyaromatic-substituted glycosides from *G. elata* have been studied for structural properties. All of them were identified in our metabolomics. Noteworthy, 1-O-(4-hydroxymethylphenoxy)-2-O-trans-cinnamoyl-β-D-glucoside, a new derivative of gastrodin with a trans-cinnamoyl unit (pos_3330) was up-accumulated in the GR group. Moreover, 2′-O-(4″-hydroxybenzyl) gastrodin was found to be more abundant in the GG than GB or GR groups. Unfortunately, there have been no pharmacological studies of these compounds to date.

Polybenzyl ethers were a class of organic compounds with at least one ether group that contained an oxygen atom connected to two benzyl groups. In 1981, bis-(4-hydroxybenzyl) ether was identified as the first polybenzyl ether in *G. elata* (Taguchi et al., 1981). To date, 21 polybenzyl ethers have been reported in the prior research (Yu H. et al., 2022). Among them, 17 members appeared in our metabolomics data (**Supplementary Table S3**). Most noteworthy, gastrols were more accumulated in the GB or GG compared with GR group. A prior study has exhibited that gastrol had relaxant effects on smooth muscle preparations isolated from the guinea pig ileum (Hayashi et al., 2002). In addition to that study, there has been few pharmacological research on polybenzyl ethers in *G. elata*.

Polybenzyl compounds are a kind of specific phenolic derivatives containing biphenylyl group, which have been widely studied in *G. elata.* In total, 31 polybenzyl compounds have been reported in previous studies, including 4,4′-dihydroxydibenzyl methane, gastrodin B, gastrol B, gastropolybenzylols, gastrodibenzins, and gastrotribenzins (**Supplementary Table S3**). One of these was 4,4′-dihydroxydibenzyl methane, a metabolite more abundant in the GG group, which was found to activate melatonin receptors (Yu H. et al., 2022). Similarly, gastropolybenzylol G, an upregulated metabolite in the GG, was shown to have agonist effects on melatonin receptors *in vitro* (Chen et al., 2019). Nevertheless, gastrodin B, an upregulated compounds in the GB and GG, has been suggested to have neuroprotective effects on H_2_O_2_-induced PC12 cell damage (Zhang et al., 2013). Plenty of studies have suggested that polybenzyl compounds from *G. elata* showed potential efficacy in improving sleep and enhancing immunity.

To date, the research on other aromatic compounds in *G. elata* is limited (**Supplementary Table S3**). Overall, eight furans, five phenylpropanoids, and one fused ring compound were isolated from this orchid (Yu H. et al., 2022). However, only a few metabolites were identified in our data, including cymbinodin A, 5-hydroxymethyl fural, 5-hydroxymethyl fural, and 5-(4-hydroxylbenzyloxymethyl)-furan-2-carbaldehyde.

### 3.4 heteroatomic compounds

Based on previous studies on *G. elata*, a variety of heteroatomic compounds such as nitrogen and sulfur compounds were isolated and detected. In the case of N-containing compounds, derivatives of amino acids and nucleosides were the main constituents reported by the previous studies (shown in **Table 1**. **Supplementary Table S3**). In more detail, gastrodamine (Zhan et al., 2016), di-(p-hydroxyl benzyl) hydroxylamine, and gastrodamine were upregulated heteroatomic aroma compounds in the GR than in the GB and GG groups. However, grossamide was not a DEM between the *G. elata* varieties groups, which was a representative lignanamide in hemp seed and possessed potential anti-neuroinflammatory actions (Luo et al., 2017). Moreover, there is a large number of nutritional and pharmacological research on nucleosides, amino acids, and their derivatives; therefore, we have not provided further information here.

**Table 1:** DEMs annotated as heteroatomic compounds, organic acids, carbohydrates, and others.

Metabolites Name	Formula	Exact Mass	Pubchem CID	CAS ID	Metabolites ID	Fold change between *G. elata* varieties group		
						GB vs. GR	GB vs. GG	GR vs. GG
N-containing compounds: nucleosides, nucleosides and etc.								
gastrodamine	C14H15NO3	245.1051			neg_559	0.0081	1.0490	128.2536
4-hydroxybenzyl guanosine	C17H19N5O6	389.1335			pos_2967	0.9513	0.2224	0.2338
N6-(4-hydroxyzenzyl) adenosine	C17H19N5O5	373.1386			pos_2751	0.9116	0.0965	0.1058
tyrosine	C9H11NO3	181.0738	6057	60-18-4	neg_207	0.1802	1.3844	7.6789
S-containing compounds: thioether, thioester, sulfonamide, sulfone, sulfoxide, sulfonic acid.								
(-)-(SS)-γ-L-glutamyl-L-[S-(4-hydroxybenzyl)] cysteinylglycine sulfoxide	C17H23N3O8S	429.1205			pos_3513	1.3599	3.5515	2.6114
S-(4-hydroxybenzyl)-glutathione	C17H23N3O7S	413.1256	10364396		neg_1885	2.2947	0.7916	0.3449
Carbohydrates and glycosides								
bis (4-hydroxybenzyl) ether mono-β-L-galactopyranoside	C20H24O8	392.1471			neg_1728	1.8058	0.1162	0.0643
Organic acids and their esters								
citric acid	C6H8O7	192.027	311	77-92-9	pos_393	2.4725	1.2711	0.5141
(E)-5,9-dihydroxydodec-6-enoic acid	C12H22O4	230.1518	129684427		neg_448	0.1645	0.6111	3.7142
6-methyl citrate	C7H10O7	206.0426	12566215	26163-65-5	pos_504	3.3025	1.1305	0.3423
trans-3-phenylacrylic acid	C9H8O2	148.0524	444539	140-10-3	neg_90	2.7504	55.8830	20.3176
dibutyl phthalate	C16H22O4	278.1518	3026	84-74-2	neg_798	0.5926	0.2093	0.3532
dimethyl phthalate	C10H10O4	194.0579	8554	131-11-3	neg_270	1.2888	4.3834	3.4010
Others								
cymbinodin A	C15H10O4	254.0579	3086629	130837-95-5	pos_1019	101.8888	9.0726	0.0890
luteolin	C15H10O6	286.04719	5280445	491-70-3	neg_867	1.6891	0.0570	0.0338

The second type of heteroatomic compounds was the S-containing compounds, including thioester, sulfonamide, sulfone, sulfoxide, and sulfonic acid (Yu H. et al., 2022). In total, more than 20 S-containing compounds were isolated from *G. elata*. Here, there were eight metabolites that were detected by UPLC-MS/MS analysis (**Table 1**; **Supplementary Table S3**). Of these, S-(4-hydroxybenzyl)-glutathione was obviously accumulated in the GB group. There was much evidence of the connection between S-containing aroma compounds and anti-cancer or anti-bacterial activities. A pharmacological study showed that (−)-(SS)-γ-L-glutamyl-L-[S-(4-hydroxybenzyl)] cysteinylglycine sulfoxide could increase the cell viability and show protective effects on serum deprivation-induced PC12 cell injury (Guo et al., 2015). Therefore, heteroatomic compounds from *G. elata* show a promising potential for further development and application.

### 3.5 Organic acids, carbohydrates, and other analysis

Organic acids and their esters were a major constituent in *G. elata.* In the present study, 11 of the 14 known organic acids were identified by our metabolomics analysis. Similar with most parishins, citric acid was more abundant in the GB than in the GR group, which was the basic element of parishins. The broad pharmacological activities of citric acid have been elaborated upon in numerous studies. It finds its application in almost all the food and pharmaceutical industry as a flavoring, acidifier, and chelating agent (Lambros et al., 2022). Additionally, trans-3-phenylacrylic acid, an upregulated metabolite in the GB or GG groups, demonstrated therapeutic effects in cancer, bacterial infections, diabetes, and neurological disorders (Ruwizhi and Aderibigbe, 2020).

In recent years, some studies have focused on carbohydrates, steroids, flavonoids, and their glycosides (Sun et al., 2023). Currently, there are nine steroids isolated from *G. elata*. Unfortunately, none of them appeared in our metabonimics data. In contrast, systematic research on flavonoids in *G. elata* has not been carried out previously, while more than 40 flavonoids have been annotated in our metabonomics analysis (**Supplementary Table S1**). Therefore, the need for extensive research on carbohydrates, steroids, flavonoids, and their glycosides in *G. elata* is highlighted.

As shown in **Figure 3B**, all DEMs were mapped to KEGG pathways, most of which were enriched in phenylpropanoid biosynthesis, isoquinoline alkaloid biosynthesis, arginine biosynthesis, flavonoid biosynthesis, phenylalanine metabolism, purine metabolism, glutathione metabolism, and pyrimidine metabolism.

### 3.6 Detection of gastrodin, 4-hydroxybenzyl alcohol, citric acid, and adenosine

**Figure 4:**
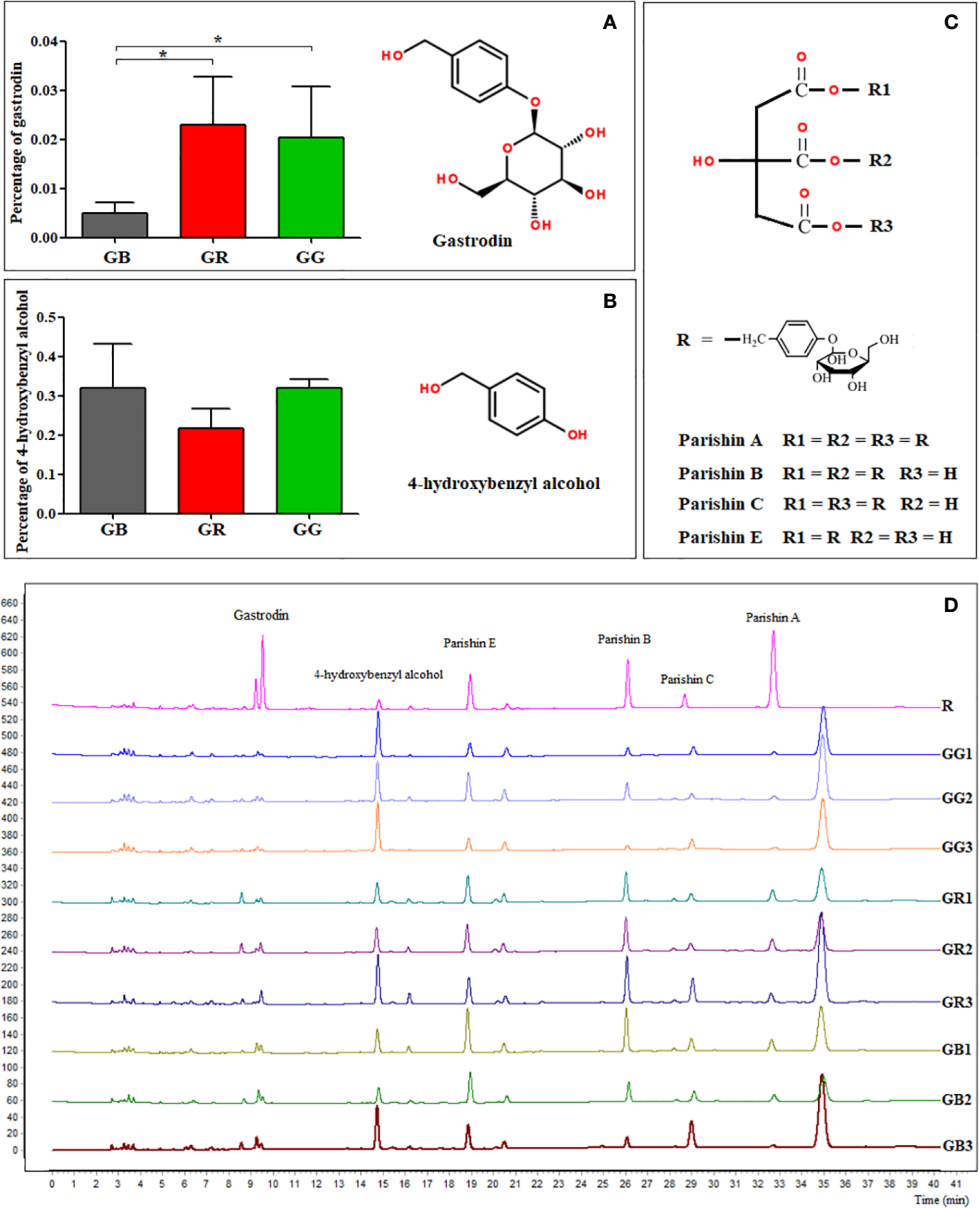
The content of gastrodin **(A)** and 4-hydroxybenzyl alcohol **(B)**, parishins structures **(C)**, and the characteristic fingerprint chromatography **(D)**. * statistically significant, p < 0.05.

Gastrodin and 4-hydroxybenzyl alcohol are widely considered to be the primary phytometabolites with medicinal functions of *G. elata* tubers. Both of them are chemical markers for quality control of Gastrodiae Rhizoma. As described in Chinese Pharmacopeia 2020 edition, the total content of gastrodin and 4-hydroxybenzyl alcohol must be higher than 0.25% in the medicinal materials of *G. elata*. In our study (**Figure 4**), the content of gastrodin in GR and GG samples were 0.02301% and 0.02039%, respectively, which were significantly higher than 0.00496% in GB samples. However, the content of 4-hydroxybenzyl alcohol in the GG, GR, and GG group were 0.3186%, 0.2456%, and 0.3203%, with no differences among three groups.

Citric acid and adenosine were differentially expressed metabolites described in our metabolomics data. Here, the determination of citric acid and adenosine with HPLC reported similar results. In GB and GB samples, the content of citric acid was 50.16 ± 6.05 mg/g and 53.72 ± 1.19 mg/g, respectively, which was significantly higher than 40.75 ± 1.79 mg/g in GR. In addition, the content of adenosine in GG (29.02 ± 0.69 μg/g) was significantly higher than that in GB (23.09 ± 1.72 μg/g) and GR (24.12 ± 1.06 μg/g). Overall, the results from HPLC analysis reported similar findings in the previous metabolomics data.

### 3.7 HPLC fingerprints

The Chinese Pharmacopeia 2020 edition and several studies have provided several practical methods for evaluating *G. elata* tubers. Among them, HPLC fingerprints is an efficient technique for the analysis of complex substances. In a previous study, HPLC fingerprint was used for analyzing the similarity and difference among Gastrodiae Rhizoma from different producing areas (Sun et al., 2019). In another study, this technique has been applied to determine the content of each active component in Gastrodiae Rhizoma (Li et al., 2019). Nevertheless, previous studies were primarily focused on the quality control of Gastrodiae Rhizoma (cut crude drugs). In this study, application of freeze-dried powder from fresh tubers in HPLC fingerprint analysis was beneficial for investigating the characteristic constituents of different *G. elata* varieties comprehensively.

In our result (**Figure 4D**), six peaks were unambiguously detected as gastrodin, 4-hydroxybenzyl alcohol, parishin E, B, C, and A, respectively, based on comparison of their chromatographic retention with that of reference standards. All of them were detected by our metabolomics data. Significantly, it has been shown that reference medicinal materials (R line, cut crude drugs) are higher in gastrodin and lower in 4-hydroxybenzyl alcohol compared with our freeze-dried samples. According to the preparation methods of Rhizoma Gastrodiae, fresh tubers are washed in water, boiled or steamed, cut into slices, and then dried, such as reference medicinal materials. Previous studies have shown that high-temperature processing such as boiling or steaming treatment could significantly increase gastrodin content (Xie et al., 2023). Thus, gastrodin content in fresh tubers is obviously lower than in cut crude drugs. As shown in our results, HPLC fingerprint technique can also be used for the quality evaluation of fresh tubers from different *G. elata* varieties. In addition, fresh samples could accurately reflect the composition difference between plant varieties.

## 4 Conclusion

In this study, we analyzed the global metabolic profiles of three *G. elata* varieties in China. Our integrated bioinformatics pipeline enabled metabolite classification and identification. Based on UPLC-MS/MS-based metabolomics, there were a total of 990 metabolites identified and annotated. The majority of DEMs were classified as aromatic compounds, heteroatomic compounds, furans, carbohydrates, organic acids, and their derivatives. In detail, parishins, vanilloloside, and gastrodin A/B were significantly higher in the GB samples, whereas gastrodin, gasrtrol, and 3,4-dihydroxybenz aldehyde were more enriched in the GR or GG. In light of our findings, numerous differential metabolites from different *G. elata* varieties implied that this orchid could have diverse biological activities and healthcare values.

## Data availability statement

The original contributions presented in the study are included in the article/**Supplementary Material**. Further inquiries can be directed to the corresponding author.

## Author contributions

XZ and SG discussed and planned the work. JL and TC conducted the experiments. XZ and YL carried out the data analysis. XZ and JL wrote the manuscript. SG revised parts of the manuscript. All authors contributed to manuscript revision and approved the submitted version.

## Supplementary material

Supplementary Table 1 Raw data from metabolomics of *G. elata*.

Supplementary Table 2 Overview of annotated metabolites.

Supplementary Table 3 Phytometabolites in *G. elata*.

Supplementary Figure 1 The standard curve of citric acid and adenosine.

Supplementary Figure 2 Typical base peak intensity chromatograms of QC samples.
