# Supplementary figures and images for: Global metabolic profile and multiple phytometabolites in the different varieties of Gastrodia elata Blume
Source: Front Plant Sci. 2023 Oct 17;14:1249456. doi: 10.3389/fpls.2023.1249456 (PMC10616830; doi:10.3389/fpls.2023.1249456)

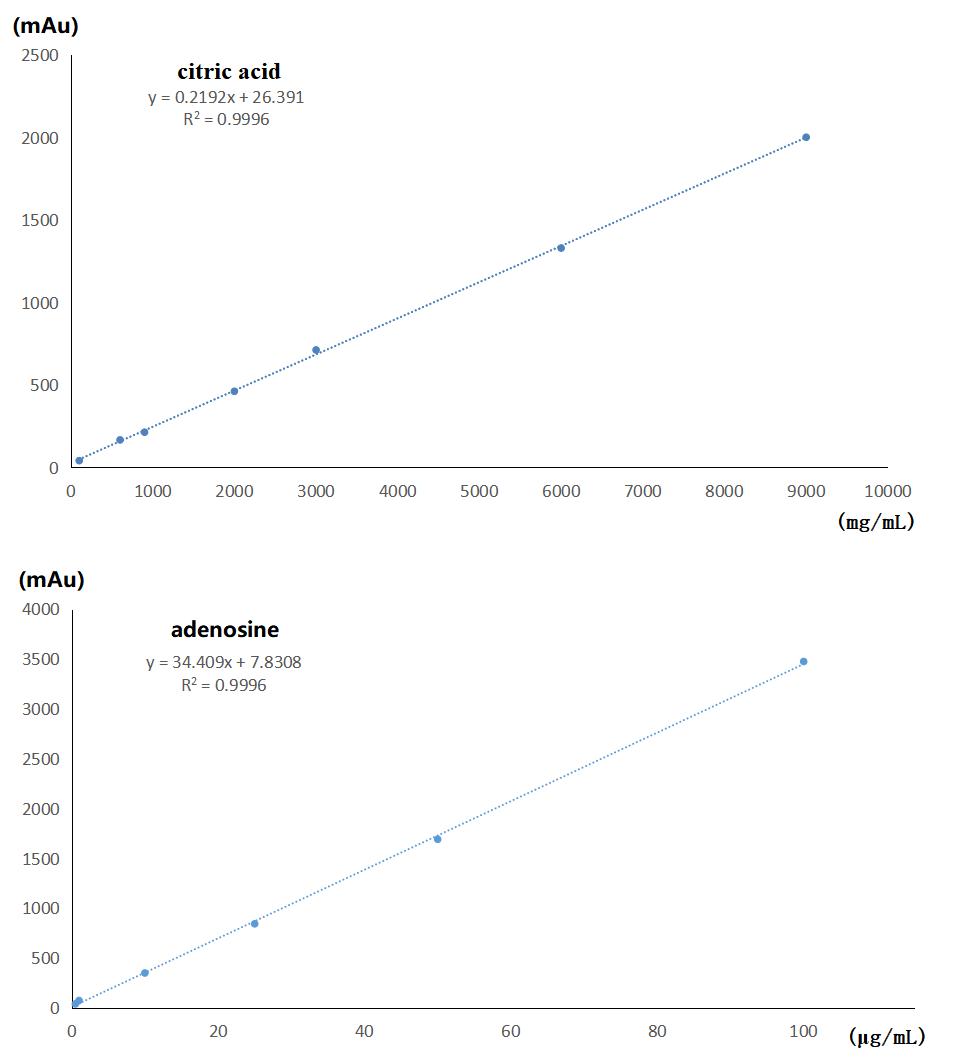

Supplement: Supplementary Figure 1 — The standard curve of citric acid and adenosine. [file Image_1.tif]

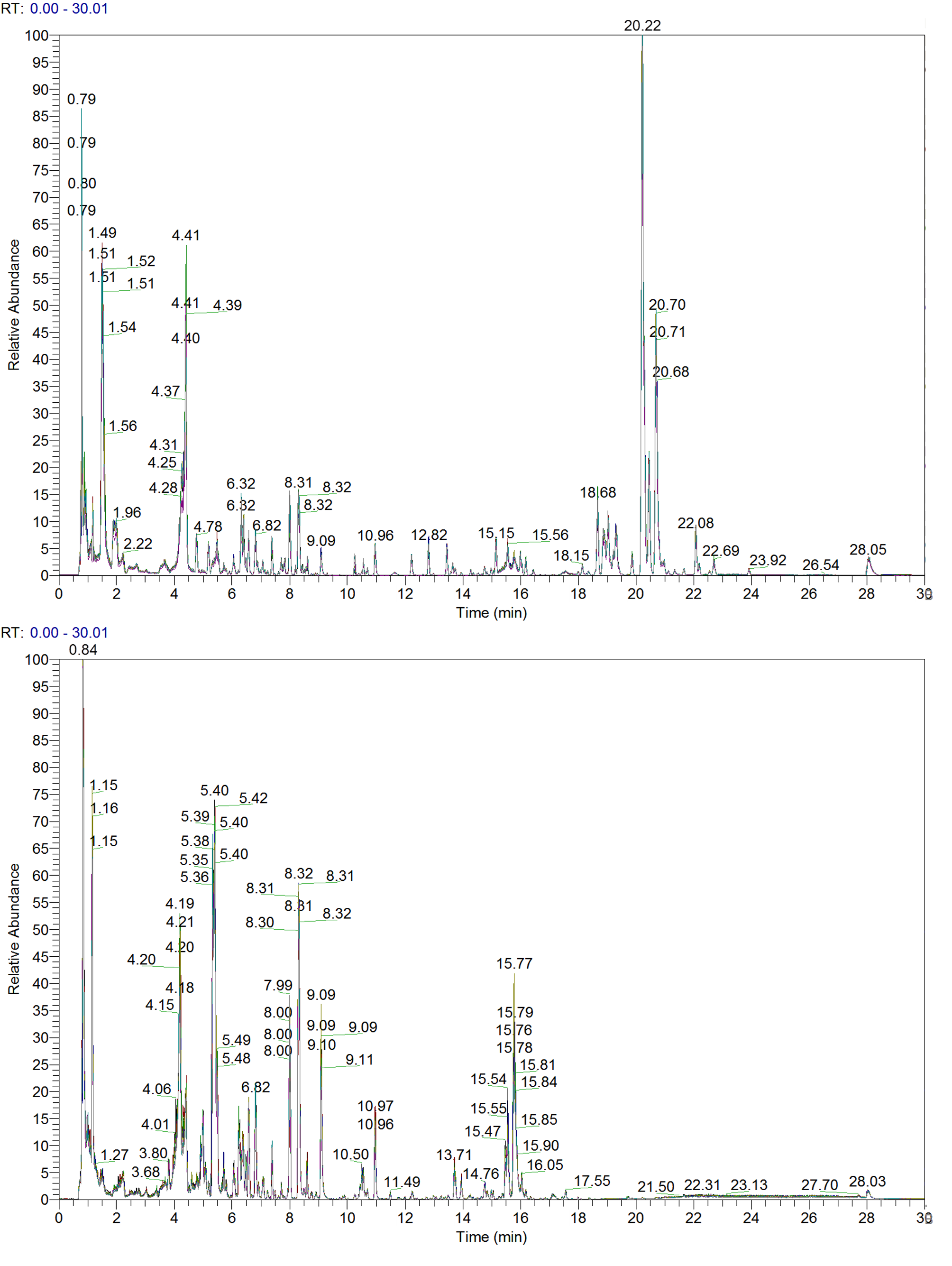

Supplement: Supplementary Figure 2 — Typical base peak intensity chromatograms of QC samples. [file Image_2.tif]
